# Self-reported meal planning practices among households in the Tshwane North area, Gauteng

**DOI:** 10.4102/hsag.v29i0.2750

**Published:** 2024-12-19

**Authors:** Lindiwe J. Ncube, Mashudu Manafe, Reno E. Gordon

**Affiliations:** 1 Division of Hospitality Management, Faculty of Economics, Development and Business Sciences, University of Mpumalanga, Mbombela, South Africa; 2 Department of Human Nutrition and Dietetics, School of Health Care Sciences, Sefako Makgatho Health Sciences University, Pretoria, South Africa

**Keywords:** menu planning, meal planning, households, food purchases, food intake

## Abstract

**Background:**

Meal planning is crucial for households to improve food choices and promote healthier eating habits.

**Aim:**

The study aims to assess meal planning practices in households in Tshwane area, Gauteng province.

**Setting:**

The study was conducted in households, north of Tshwane, Gauteng province.

**Methods:**

A survey questionnaire was administered to 368 households. Descriptive statistics were analysed, and Pearson’s chi-square test was used to assess the relationship between categorical variables. The Spearman-Rho correlation coefficient was used to determine the relationship between variables. A *p* ≤ 0.05 was considered statistically significant and a Spearman Rho correlation coefficient (*r*) ≥ 0.25 signified a positive relationship.

**Results:**

Sixty-one per cent of the participants regarded meal planning as important. A total of 137 participants (37%) reported that they planned their meals before cooking and 64% (*n* = 235) never used recipes. There was statistical significance (*p* <0.05) between the options considered when buying food and the age of the participants. A positive association (*p* < 0.05) between the options considered when planning meals and employment status were obtained. The Spearman’s Rho correlation coefficient showed a positive relationship between the options considered when planning meals and buying groceries (*r* = 0.377, *p* < 0.001).

**Conclusion:**

Meal planning was considered important by participants but was not fully practised in households. Therefore, interventions through practical sessions are recommended to improve household’s meal planning and meal preparation practices, including household’s cooking skills.

**Contribution:**

Community-based nutrition education intervention strategies will empower households to opt for healthier meals through meal planning.

## Introduction

Many developing countries, including South Africa, are experiencing a transition in dietary practices such as a shift away from traditional foods to processed foods high in sugar, fat and sodium, in addition to an increased sedentary lifestyle. This transition is because of increased industrialisation and urbanisation and has resulted in the double burden of malnutrition. Consumption of foods high in sugars, saturated and trans fats, low fibre contributes to an increased risk of overweight, obesity and non-communicable diseases (Madlala et al. 2022). Ducrot et al. (2017) suggested that meal planning is a potential tool to compensate for time limitations, which is seen with the demands of the modern lifestyle. Meal planning encourages the preparation of meals at home, which is associated with improved diet quality and choices of various food items.

Households that plan meals are likely to consume healthier meals than those who buy and prepare foods impulsively with little or no planning (Crawford et al. 2007; Patch, Tapsell & Williams 2005). Those who plan their meals are more likely to have better diet quality, as they are likely to adhere to nutritional guidelines (Fernandez et al. 2020). A study by Fulkerson et al. (2011) reported that urban households in Minneapolis were keen to have meal planning programmes. However, African-American households were found to be inconsistent in family meal planning and participate in fewer family meals per week (Fruh et al. 2013). Households with reduced meal pre-planning practices are less likely to participate in family meals (Ducrot et al. 2017). Additionally, households reported a need for creative ways to help them plan and prepare healthy meals quickly. Well-planned family meals lead to better nutrition, including more fruits, vegetables, grains, calcium-rich foods, protein, iron, fibres and vitamins A, C, E, B_6_ and folate. They also expose children to a wider variety of foods and reduce the intake of soft drinks and snack foods. Children who eat with their families have improved vocabulary and reading skills and learn social skills such as table manners (Fiese & Schwartz 2008). As these children grow older, they engage in fewer risk-taking behaviours. Therefore, household meal planning activities are important to ensure that families choose and consume healthy and balanced meals. However, household meal planning practices have received little attention in the scientific literature. Planning meals may reduce the risk of missing ingredients for home meal preparation, which could also lead to consuming food prepared away from home (Ducrot et al. 2017). Although these studies are all international, they denote a need for improving household’s meal planning skills, which leads to healthier meal consumption, efficient buying and the use of food ingredients. Healthy eating is also needed in households in sub-Saharan Africa, including South Africa as it improves the household’s nutritional and health status.

Food choice is a complicated and complex phenomenon prone to change over time. Food choices also influence household’s activities positively and negatively. A better understanding of food choices and the importance for targeted communities may inform strategies to improve diets, nutrition, health outcomes and overall well-being (Sobal & Bisogni 2009). However, consumers are less likely to choose unhealthy foods because of cost, convenience advantages and ignorance. This makes the choices between taste and nutrition more apparent (Binkley & Golub 2011). Consuming healthier diets by following national dietary guidelines is achievable with reasonable food choices without affecting the meal plan cost (Kh’ng, Chang & Hsu 2022). Nonetheless, the cost associated with a healthy meal plan is still likely to be a barrier to the low-income population.

Black households in Gauteng consumed more indigenous foods such as sorghum, marula, pearl millet, amadumbe and cowpea for nutritional and health reasons, including availability (Kesa et al. 2023). Similarly, in a study conducted in urban South Africa, food availability, resources and economic factors were the most important for lower-income (LI) households. Whereas health, familiarity and food exploration were mostly mentioned by middle-income (MI) and high-income (HI) households (Magano, Tuorila & De Kock 2023). De Bem Lignani et al. (2011) reported that the increase in the purchasing power of poor families increased the household’s unhealthy food choices. Healthy eating behaviours are generally poor among food-insecure households because of factors including perceived higher cost of healthy foods, financial stress, inadequate nutritional meal planning knowledge and inadequate skills required for healthy food preparation (Van der Velde et al. 2019). Although this is an old study, Binkley and Golub (2011) found that the presence of children in a household negatively influenced healthy food choices although households with older members made healthier choices.

The World Health Organization (2016) suggests that preferences and eating behaviours are established in early life. Early food deprivation in childhood and associated preferences, including food consumption behaviours, influence childhood poverty and adult obesity (Leifheit et al. 2020). Food deprivation and irregular availability of healthy foods during childhood led to poor eating behaviours and less healthy food choices (Scaglioni et al. 2018). Meal planning was associated with lower odds of being overweight and obese (Fernandez et al. 2020).

Meal planning practices and food choices are strongly influenced by the social and cultural preferences of individuals and households. Food intake affects health status; however, most households consider personal choice more imperative than nutritional value (Food and Agriculture Organization of the United Nations et al. 2019).

A study conducted by Ariani et al. (2021) in Indonesia showed that food consumption behaviour was not balanced regarding tubers, legumes, animal protein sources, fruits and vegetables. Therefore, in HI households, the consumption of animal products was high. During coronavirus disease 2019 (COVID-19), there was an increase in the consumption of immune-boosting foods, traditional herbs and spices among middle-class households in Bengaluru, India. Furthermore, there was no change in food consumption before and after COVID-19. However, there was a change in purchasing behaviours such as online, restaurant and supermarket purchases among Hispanic and non-Hispanic black households (Antwi et al. 2023). In a study conducted in Brazil, there was an increase in the consumption of cereals, processed foods, meat and sugar. The authors stated that the purchasing power increase among poor families increased the household’s unhealthy food choices (De Bem Lignani et al. 2011).

South Africa, like other developing and underdeveloped countries, is undergoing rapid urbanisation that is closely associated with various social structural changes, including migration, modernisation, globalisation, economic advancement and enculturation. Instantaneously, food systems and circumstances undergo major changes because of technological advancements, food policies and changes in consumer lifestyle. The urban environment also appears to negatively influence food choices, preferences and household food consumption behaviours, where the majority of urban South African households’ behaviours are associated with high consumption of energy, salt, saturated fat and refined sugars with a low fruit, vegetables and fibre consumption. Much attention focuses on how the urban food environment influences and shapes consumer food consumption and subsequent health (Casari et al. 2022; Colozza, Wang & Avendano 2023; Westbury et al. 2021). Numerous studies investigated meal planning practices, food choices and food consumption behaviours among households (Landry et al. 2020; Moffat et al. 2021; Ranjit, Macias & Hoelscher 2020); however, there is a dearth of sub-Saharan studies carried out on these phenomena. Thus, this study aims to assess meal planning practices in households in the Tshwane area, Gauteng province, South Africa.

## Research methods and design

### Study design

A quantitative descriptive study design was used to describe the self-reported meal planning practices, food choices and food consumption behaviours of households in the north of Tshwane, Gauteng province.

### Study population and sampling strategy

This study was conducted in an urban township 37 km north of Tshwane in Gauteng province and the target population was 7 700 households. The online Raosoft sample size calculator was used to calculate the sample size with a 5% margin of error, 95% confidence interval and 50% response distribution, from this a sample size of 368 was obtained. Probability systematic sampling was used to select households from a list of all households obtained from the area municipality (Leedy & Ormrod 2016). Every fifth house was selected until the sample size of 368 households was met.

### Data collection

A researcher-administered questionnaire comprising three sections was used: Section A assessed socio-demographic characteristics, section B assessed planning and organising for meal preparation and section C looked at household choices and food choices. These sections were extracted from a larger questionnaire that was previously used and validated by Nesamvuni (2014). In addition, the three sections were also piloted among ten households in the same area where the main study was conducted. The pilot study helped to identify flaws in the questionnaire and assessed if each questionnaire captured the information it intended to. Households who participated in the pilot study were not considered for the main study. Before data collection, four field workers were trained on the content of the questionnaire and surveying techniques. Field workers collected data from an adult household member responsible for cooking in the house. In cases where no one was available at home, the field worker collected data from the next household, the number was documented, and additional households were interviewed as replacements. After the interview, each questionnaire was checked for completeness.

### Reliability

The study methods were consistently applied, and data collection procedures were standardised by training field workers before the data collection process. The data collection tool was pretested before the main study.

### Validity

The questionnaire used in this study was designed by adapting some questions from a previously used questionnaire by Nesamvuni (2014), and the questionnaire was reviewed by experts to ensure face validity and piloted as detailed above.

### Data analysis

Data were coded, captured on a Microsoft Excel spreadsheet and imported to STATA version 13 statistical software (StataCorp, LLC, College Station, TX, US) for analysis. Descriptive statistics such as frequencies, mean and standard deviation were analysed. Logistic regression analysis was also performed to assess the relationships between demographic variables, meal-planning practices and household food choices. To assess the relationship between meal planning practices and household food choices the Spearman’s Rho correlation coefficient was used. The correlations are significant where Spearman’s Rho (*r*) < 0.25 and *p* ≤ 0.05.

### Ethical considerations

The study was carried out according to the Declaration of Helsinki, and the protocol was approved by the Sefako Makgatho University Research Ethics Committee (SMUREC) (certificate no. SMUREC/H/233/2016: IR). Permission to conduct the study was obtained from the municipal manager. All participants gave their informed consent before participating in the study. Informed consent was obtained from the participants in the local language (Setswana). The consent form was translated into Setswana and explained to the participants to ensure that those with low literacy levels and who could not understand English were not disadvantaged.

## Results

The sample comprised 70% (*n* = 258) females and 28% (*n* = 110) males. More than a third of the participants (*n* = 137, 37%) were between the ages of 35 and 55 years (*n* = 137, 37%), and in 144 (39%) households, only one adult was employed. Most of the participants (*n* = 235, 64%) had secondary education (Table 1).

**TABLE 1:** Demographic characteristics (*N* = 368).

Variables	Number (*n*)	%
**Age (years)**		
18–34	90	24
35–55	137	37
56–64	52	14
65 and above	89	24
**Gender**		
Male	110	30
Female	258	70
**Number of adults employed**		
No adult employed	141	38
One adult employed	144	39
Two or more adults employed	83	33
**Education level**		
No primary education	5	1
Primary education	71	19
Secondary education	235	64
Tertiary education	57	15

### Planning and organising meals

**FIGURE 1:**
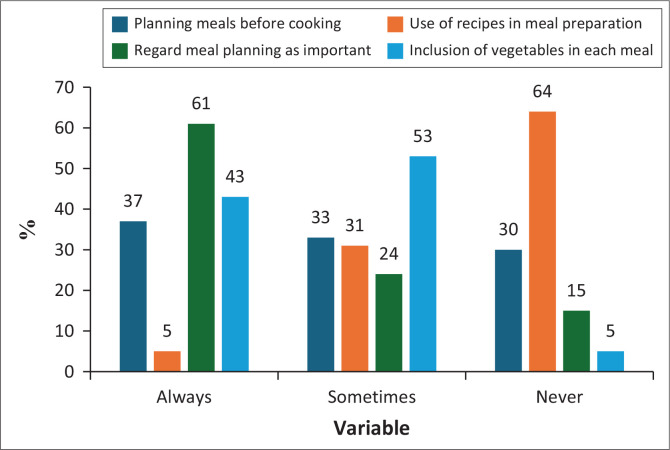
Planning and organising meals.

As Figure 1 shows, although meal planning was regarded as important by most participants (61%), there was poor practice of not planning meals and never using recipes in meal preparation.

### Self-reported meal planning practices and gender

Table 2 shows that there is an influence on self-reported meal planning practices with the type of food eaten daily (*p* < 0.05), the type of meat preferred in the home and the gender of the respondents (*p* < 0.05).

**TABLE 2:** Relationship between self-reported meal planning practices and gender.

Variables	Male		Female		*p*
	*n*	%	*n*	%	
**Options considered when planning meals**	-	-	-	-	0.299
Family food preferences	24	22	57	22	-
Availability	47	43	118	46	-
Season	5	5	3	1	-
Budget	8	7	23	9	-
Nutritional needs	3	3	3	1	-
Schedules	1	1	2	1	-
Convenience in cooking	0	0	6	2	-
Other	22	20	46	18	-
**Options considered when buying groceries**	-	-	-	-	0.857
Menu planned	13	12	34	13	-
Food items you want to eat	47	43	120	47	-
Grocery store special	33	30	65	25	-
Nutrient content	2	2	3	1	-
All options	15	14	34	13	-
**Type of food eaten daily**	-	-	-	-	0.004*
Fast foods	5	5	7	3	-
Fried foods	12	11	9	3	-
Prepared foods	75	68	215	83	-
Hamburger	0	0	1	0	-
Traditional foods	1	1	7	3	-
All types	17	15	19	7	-
**Type of meat prefer to buy**	-	-	-	-	0.032*
Fish	11	10	18	7	-
Chicken	51	46	87	34	-
Red meat	16	15	47	18	-
Chicken and red meat	31	28	106	41	-
Fish and chicken	1	1	0	0	-

* , indicates that there is a statistical significance at *p* < 0.05.

### Relationship between self-reported meal planning practices and age

There was statistical significance between self-reported meal planning practices such as the options considered when purchasing groceries (*p* < 0.05), type of food eaten daily (*p* < 0.05) and age (Table 3).

**TABLE 3:** Relationship between self-reported meal planning practices and age.

Variables	Age (years)								*p*
	18–34		35–55		56–64		65+		
	*n*	%	*n*	%	*n*	%	*n*	%	
**Options considered when planning meals**	-	-	-	-	-	-	-	-	0.736
Family food preferences	24	27	26	19	12	23	19	21	-
Availability	43	48	61	45	27	52	34	38	-
Season	1	1	2	1	2	4	3	3	-
Budget	6	7	13	9	3	6	9	10	-
Nutritional needs	0	0	2	1	0	0	4	4	-
Schedules	1	1	1	1	0	0	1	1	-
Convenience in cooking	1	1	3	2	0	0	2	2	-
Other	14	16	29	21	8	15	17	19	-
**Options considered while buying groceries**	-	-	-	-	-	-	-	-	0.004*
Menu planned	11	12	16	12	3	6	17	19	-
Food items you want to eat	46	52	49	36	29	56	44	49	-
Grocery store special	22	25	40	29	12	23	24	27	-
Nutrient content	0	0	5	4	0	0	0	0	-
All options	10	11	27	20	8	15	4	4	-
**Type of food eaten daily**	-	-	-	-	-	-	-	-	0.001*
Fast foods	7	8	2	1	2	4	1	1	-
Fried foods	2	2	12	9	4	8	3	3	-
Prepared foods	63	70	101	74	43	83	83	93	-
Hamburger	1	1	0	0	0	0	0	0	-
Traditional foods	5	6	2	1	1	2	0	0	-
All types	12	13	20	15	2	4	2	2	-
**Type of meat prefer to buy**	-	-	-	-	-	-	-	-	0.215
Fish	11	12	7	5	5	10	6	7	-
Chicken	38	42	46	34	21	40	33	37	-
Red meat	16	18	20	15	9	17	18	20	-
Chicken and red meat	24	27	64	47	17	33	32	36	-
Fish and chicken	1	1	0	0	0	0	0	0	-

* , indicates that there is a statistical significance at *p* < 0.05.

### Relationship between self-reported meal planning and employment status

As Table 4 shows, there was a positive association between the options considered when planning meals (*p* < 0.05) and the employment status, especially where a member of the household is employed and dependent on the food items the household wants to include in the meal.

**TABLE 4:** Relationship between self-reported meal planning practices and employment status.

Variables	Employment status of household members						*p*
	None employed		One employed		Two or more employed		
	*n*	%	*n*	%	*n*	%	
**Options considered when planning meals**	-	-	-	-	-	-	0.01*
Family food preferences	29	36	26	32	26	32	-
Availability	62	38	65	39	38	23	-
Season	5	63	2	25	1	12	-
Budget	16	51	12	39	3	10	-
Nutritional needs	1	17	2	33	23	50	-
Schedules	0	0	3	100	0	0	-
Convenience in cooking	0	0	6	100	0	0	-
Other	28	41	28	41	12	18	-
**Options considered when buying groceries**	-	-	-	-	-	-	0.06
Menu planned	26	55	14	30	7	15	-
Food items you want to eat	50	30	73	43	45	27	-
Grocery store special	43	44	38	39	17	17	-
Nutrient content	2	40	1	20	2	40	-
All options	20	41	17	35	12	24	-
**Type of food eaten daily**	-	-	-	-	-	-	0.06
Fast foods	3	25	3	25	5	50	-
Fried foods	9	4	7	33	5	24	-
Prepared foods	118	41	110	38	62	21	-
Hamburger	0	0	1	100	0	0	-
Traditional foods	1	12	7	88	0	0	-
All types	10	28	16	44	10	28	-
**Type of meat prefer to buy**	-	-	-	-	-	-	0.14
Fish	11	38	10	34	8	28	-
Chicken	50	36	62	45	26	19	-
Red meat	25	40	16	25	22	35	-
Chicken and red meat	54	39	56	41	27	20	-
Fish and chicken	1	100	0	0	0	0	-

* , indicates that there is a statistical significance at *p* < 0.05.

### Spearman’s rho correlation coefficients of factor variables

A Spearman’s rank correlation coefficient was computed to determine the relationship between meal planning and food choice consumption behaviour (See Table 5). The results indicated a positive relationship between the options considered when planning meals and buying groceries (*r* =0.37, [*p* < 0.05]). The results also showed a positive relationship between the importance of meal planning and the behaviour of cooking meal planning, (*r* = 0.34, [*p* < 0.05]). A positive correlation was observed between the type of foods eaten daily, options considered when planning meals and options considered when buying groceries (*p* < 0.05). In addition, the inclusion of vegetables in each meal was positively correlated with the options considered when planning meals (*p* < 0.05) and the options when buying groceries (*p* < 0.05).

**TABLE 5:** Results of Spearman’s Rho correlation coefficients of factor variables.

Variables	Planning meals before cooking	Options considered when planning meals	Use recipe in meal preparation	Options when buying groceries	Meal planning importance	Type of food eaten daily	Inclusion of vegetables in each meal
**Planning meals before cooking**							
*r*	1.000	-	-	-	-	-	-
**Options considered when planning meals**							
*r*	0.000	1.000	-	-	-	-	-
*p*	0.889	-	-	-	-	-	-
**Use of recipes in meal preparation**							
*r*	0.000	0.090	1.000	-	-	-	-
*p*	0.943	0.070	-	-	-	-	-
**Options when buying groceries**							
*r*	0.040	0.370*	0.000	1.000	-	-	-
*p*	0.443	0.000	0.859	-	-	-	-
**Meal planning importance.**							
*r*	0.340*	−0.080	−0.020	0.020	1.000	-	-
*p*	0.000	0.126	0.662	0.583	-	-	-
**Type of food eaten daily**							
*r*	−0.030	0.230**	0.040	0.240**	−0.090	1.000	-
*p*	0.554	0.000	0.421	0.000	0.065	-	-
**Inclusion of vegetables in each meal**							
*r*	0.010	0.110**	−0.010	0.110**	−0.050	0.040	1.000
*p*	0.738	0.030	0.787	0.022	0.299	0.369	-

* , Spearman’s Rho Correlation Coefficients (*r*) ≥ 0.25 signified a positive relationship;

** , Correlation is significant at *p* < 0.05.

## Discussion

This study assessed meal planning practices in households and found that most households considered meal planning important, even though fewer people reported that they always plan meals before cooking. Although Ducrot et al. (2017) did not report how many households plan their meals before cooking, the authors found that households that used meal-planning strategies ate healthier diets compared to those who purchased and prepared foods on impulse or with little or no planning. A lack of nutrition knowledge including proper meal planning was one of the barriers to healthy eating (Amore, Buchthal & Banna 2019). Those who plan their meals have a better-quality diet because of adherence to nutritional guidelines and food variety (Fernandez et al. 2020). A lack of meal planning can be attributed to a lack of knowledge and skills (Landry et al. 2020). Meal planning can save households unnecessary expenditures as only food items required for meals will be bought, and it can reduce unplanned trips to buy one or two items, a potential tool to encourage home meal preparation, which is linked to improved diet quality. Family-based strategies are important for food literacy, where households are empowered and encouraged to prepare healthier and varied meals. The results of Spearman’s Rho correlation coefficients showed a positive relationship between the options considered when planning meals and the options considered when buying groceries. The results also showed a positive relationship between the importance of meal planning, especially before cooking.

It was interesting to notice that most households reported that they never used recipes in meal preparation. Recipes are derived from a meal plan. The use of meal plans and recipes is recommended as it allows for the identification of the ingredients and products needed to prepare meals, buying groceries for a month – which can be a huge money saver and buying only grocery items needed to produce planned meals (Ducrot et al. 2017). Meal planning can also help with organising, time management and creating a grocery list (Landry et al. 2020). Deciding what foods will be eaten in the next few days could enable individuals to cook more diversified recipes and anticipate grocery shopping for the specific ingredients needed, thus potentially explaining the increased food variety observed in meal planners (Ducrot et al. 2017). Targeted households training at community centres are therefore important to encourage related skills such as cost-effective food shopping, efficient time and resources cooking and menu planning.

Most households in this study indicated that they opt for available food items and few indicated that they choose family food preferences when planning meals. Individuals consider what to eat according to what they want to eat without considering healthy eating (Whitelock & Ensaff 2018). Consumers choose foods based on their household’s preferences and available resources (Burchi & De Muro 2016). Furthermore, previous research indicated that households choose food based on cost, time expenditures to purchase, prepare and consume food and cleaning up after preparation and consumption, including the source of income that can be used to buy food (Achón et al. 2017). In this study, participants’ age influenced options considered (food availability) when planning meals. This is because individuals in the same age group are responsible for food shopping (Shim, Hwang & Kim 2019). Equally, it was considered a common practice and behaviour in a study conducted in Spain where people compared prices and looked for discounts before buying food items (Achón et al. 2017).

Most households in this study indicated that they buy food items they want to eat, and a significant number said that they buy items on grocery store specials. Congruently, the Food and Agriculture Organization of the United Nations et al. (2019) reported that most households consider personal choice more imperative than nutritional value. Households purchase foods based on their preferences and subject to the constraint that the food cost was less than or equal to the sum of all sources of income (Burchi & De Muro 2016). Consumers are unlikely to choose unhealthy foods because of cost, convenience advantages and ignorance (Binkley & Golub 2011).

Most of the households in this study indicated that they eat home-prepared food daily and the least number said that they eat fried food daily. Households that consume home-cooked meals more than five times a week consume more vegetables and fruits daily (Mills et al. 2017). However, it might not be the case with the households in this study as the inclusion of vegetables was based on the household-planned meals and was included as part of their grocery list. Households that eat home-cooked meals more than five times a week were less likely to be overweight and obese (Mills et al. 2017). Eating one fast food meal per week in households was associated with a higher prevalence of overweight and obesity (Taillie & Poti 2017). An increase in family breakfasts and dinner frequency was associated with a lower likelihood of being overweight and obese (Mahmood et al. 2023). A significant association between time spent preparing meals and employment status, where households spending the least time on food preparation tended to be working adults prioritising convenience instead of using recipes (Monsivais, Aggarwal & Drewnowski 2014). In a study by Smith et al. (2016), conducted among Mexican Americans in the US, participants believed that food prepared at home is healthier than any other food. However, home food preparation among Mexican Americans in the US was higher among those with lower income, unemployed and a higher age. More time spent on home food preparation is associated with good diet quality, including a significantly higher intake of fruits and vegetables. The participants’ gender in this study strongly influenced the type of food eaten. In addition, participants’ age and gender influenced the choice of prepared foods in this study. The influence of gender and food preparation is based on the existing social norms that women and girls are more likely to be involved in cooking (Mills et al. 2017). Farmer et al. (2019), in a study among non-Hispanic black women, reported that they do most of the cooking. Home-cooked meals depend on the available food items, food selection and the decision on what to cook for the particular meal. Hence, most of the time, home cooking does not equal healthy cooking. Income status may influence the types of food being prepared (Coleman-Jensen et al. 2019). Other authors identified price, availability of quality items, the kind of food prepared at home, food choices and food preparation methods as barriers that could negatively influence the diet quality of food prepared at home (Wolfson et al. 2019).

In this study, most prefer to buy chicken with a significant amount stating that they buy both chicken and red meat. On the contrary, Whitelock and Ensaff (2018) reported reduced meat consumption and a growing preference for fish. It is observed that the elderly study population may have influenced the food choices (Whitelock & Ensaff 2018). Meal planning influenced vegetable inclusion in each meal, with the options considered when planning menus and buying groceries Insufficient vegetable availability and consumption were reported among food-insecure families, with the availability of vegetables in a household not translating to consumption (Canella et al. 2018). Cost is a major barrier to accessing and purchasing fresh vegetables, especially among low-income individuals (Mook et al., 2016). Women who plan meals before going to the store have a higher chance of consuming more vegetables (Fernandez et al. 2020). Encouraging people to have home gardens can promote vegetable consumption. This would be possible for this study population as they have land in their yards to make a small home garden.

### Strengths and limitations

One of the study’s strengths is using a validated questionnaire that had been used in previous research and was adapted for this study. In addition, after the pilot study, the questions were rephrased to eliminate any ambiguity. The study was conducted in an area in Tshwane and can be replicated in other areas. However, there is a likelihood of over, under or misreporting findings in self-reported studies, which serves as a limitation. Participants’ responses may be skewed because of their socially desirable ability to provide preferred answers more than real experiences (Grimm 2010).

### Implication of the study

This study identified a knowledge gap in nutritional meal planning, food choices and household food consumption. In addition, community-based nutrition intervention strategies are needed to address identified gaps and healthy eating tailored to empower and educate communities with healthy meal planning knowledge. This may influence a change in food consumption behaviours. Consequently, overweight and obesity in both children and adults may be alleviated and in the long term contribute to reducing the prevalence of food insecurity.

## Conclusion

The findings contribute to a better understanding of household meal and meal planning practices in the Tshwane North area. Therefore, the researchers suggest that further studies should be conducted on a larger scale in urban and rural settings. Interventions should focus on practical sessions to increase self-efficacy in meal preparation and cooking skills. It is important to highlight the importance of planning and using recipes and grocery lists. More research is needed to explore these parameters in other diverse populations to understand meal planning interpretations and integrate these strategies with interventions to evaluate their effectiveness in increasing the implementation of meal planning. Subsequently, this will in turn improve the diet quality of households.
